# Correlation between sequence conservation and structural thermodynamics of microRNA precursors from human, mouse, and chicken genomes

**DOI:** 10.1186/1471-2148-10-329

**Published:** 2010-10-27

**Authors:** Ming Ni, Wenjie Shu, Xiaochen Bo, Shengqi Wang, Songgang Li

**Affiliations:** 1 Center for Bioinformatics, National Laboratory of Protein Engineering and Plant Genetic Engineering, College of Life Sciences, Peking University, Beijing 100871, China; 2 Beijing Institute of Radiation Medicine, Beijing 100850, China

## Abstract

**Background:**

Previous studies have shown that microRNA precursors (pre-miRNAs) have considerably more stable secondary structures than other native RNAs (tRNA, rRNA, and mRNA) and artificial RNA sequences. However, pre-miRNAs with ultra stable secondary structures have not been investigated. It is not known if there is a tendency in pre-miRNA sequences towards or against ultra stable structures? Furthermore, the relationship between the structural thermodynamic stability of pre-miRNA and their evolution remains unclear.

**Results:**

We investigated the correlation between pre-miRNA sequence conservation and structural stability as measured by adjusted minimum folding free energies in pre-miRNAs isolated from human, mouse, and chicken. The analysis revealed that conserved and non-conserved pre-miRNA sequences had structures with similar average stabilities. However, the relatively ultra stable and unstable pre-miRNAs were more likely to be non-conserved than pre-miRNAs with moderate stability. Non-conserved pre-miRNAs had more G+C than A+U nucleotides, while conserved pre-miRNAs contained more A+U nucleotides. Notably, the U content of conserved pre-miRNAs was especially higher than that of non-conserved pre-miRNAs. Further investigations showed that conserved and non-conserved pre-miRNAs exhibited different structural element features, even though they had comparable levels of stability.

**Conclusions:**

We proposed that there is a correlation between structural thermodynamic stability and sequence conservation for pre-miRNAs from human, mouse, and chicken genomes. Our analyses suggested that pre-miRNAs with relatively ultra stable or unstable structures were less favoured by natural selection than those with moderately stable structures. Comparison of nucleotide compositions between non-conserved and conserved pre-miRNAs indicated the importance of U nucleotides in the pre-miRNA evolutionary process. Several characteristic structural elements were also detected in conserved pre-miRNAs.

## Background

MicroRNAs (miRNAs) are small endogenous non-coding RNAs that regulate expression at the post-transcriptional level in animals and plants [1]. Both plant and animal miRNAs are cleaved from one arm of foldback precursors (pre-miRNAs). It is generally accepted that pre-miRNA secondary and/or tertiary structures are critical in miRNA biogenesis [2-5]. The thermodynamic stability of pre-miRNA hairpin secondary structures, hereafter called pre-miRNA stability, is a fundamental property of RNA structure and has been systematically studied. Bonnet et al. reported that in five animal species and one plant species pre-miRNAs have significantly lower estimated folding minimum free energies (MFEs) than those of their shuffled sequences, unlike other kinds of RNAs such as tRNAs, rRNAs [6], and mRNAs [6,7]. Zhang et al. directly compared the stability of pre-miRNAs and other kinds of RNAs in seven plant species and showed that pre-miRNAs form more stable secondary structures [8]. Currently, the lower limit of pre-miRNA thermodynamic stability is widely used as a criterion for predicting and verifying sequences of RNA that constitute pre-miRNA [9-12].

However, several studies have characterized pre-miRNA sequence and structural features that can lead to pre-miRNA instability. The unwinding of pre-miRNA foldback duplex structure is critical for processing of pre-miRNAs [13,14]. Therefore, less stable pre-miRNAs may be processed more easily. Pre-miRNAs have higher total adenine (A) and uracil (U) contents than other kinds of RNAs [8]. As A-U and guanine (G)-cytosine (C) form two and three hydrogen bonds respectively, pre-miRNAs with a higher A+U content may be less stable. Some studies of miRNA biogenesis in animal species have suggested that instability, or enhanced flexibility of pre-miRNAs, resulting from mismatched nucleotides, bulges, and especially unstable base pairs at the 5' end, can increase the efficiency of Dicer enzymes involved miRNA biogenesis [15-17]. The human nuclear processing enzyme Drosha has also been found to selectively cleave pre-miRNAs hairpins bearing a large terminal loop (≥ 10 nucleotides) [18]. This type of terminal loop could also result in pre-miRNA instability. While these studies provided evidence for structural and sequence features that destabilize pre-miRNAs, a systematic investigation of pre-miRNA instability has not been carried out. It has been shown that there is a tendency against unstable pre-miRNA structures [6,8]. However, the question remains: is there a tendency against ultra stable secondary structures in pre-miRNA sequences?

A correlation between pre-miRNA stability and nucleotide sequence conservation would be expected due to natural selection, if there is a range of stability that directly or indirectly results in efficient pre-miRNA functioning. MiRNAs were previously regarded as highly conserved [1], but a number of non-conserved miRNAs have been recently found in closely related species [1,9,19-23]. To investigate the relationship between sequence conservation and thermodynamic stability, we compared the thermodynamic stability of conserved and non-conserved pre-miRNAs from human, mouse, and chicken genomes, with special emphasis on ultra stable and unstable pre-miRNA sequences. We also investigated the correlation between pre-miRNA structural elements and sequence conservation.

## Results

### Stability comparison between conserved and non-conserved pre-miRNAs

**Figure 1:**
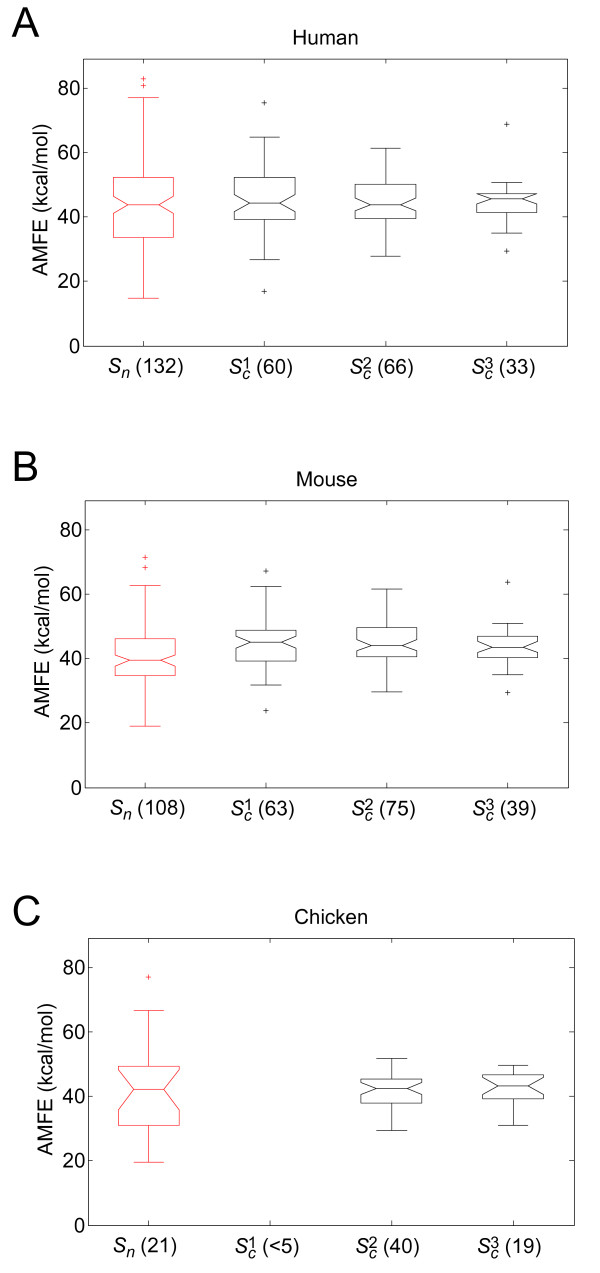
**Distribution of AMFEs**. Boxplot of AMFEs for non-conserved (*S_n_*, red) and conserved pre-miRNAs (S*_c_*^1^, S*_c_*^2^, and S*_c_*^3^, black) from human (A), mouse (B), and chicken genomes (C). The number of pre-miRNAs within each set is indicated in brackets. *S_c_*^1 ^for chicken was excluded as it contained fewer than five pre-miRNAs.

We calculated the minimum free energy (MFE) values of 658 conserved and non-conserved pre-miRNAs. As the MFEs increased linearly with sequence length, we normalized the AMFE values to 100 nucleotide sequence to yield adjusted MFEs (AMFEs) [8] for comparing pre-miRNA thermodynamic stability (Additional file 1). Potential artefacts arising from difference sequence lengths were excluded by using AMFEs for comparisons. Conserved pre-miRNAs were divided into three conservation levels, termed as *S_c_*^1^, *S_c_*^2^, and *S_c_*^3^, representing low, moderate, and high levels of conservation, respectively (see Methods). Non-conserved pre-miRNAs were classed as *S_n_*. The summary of the pre-miRNA stability, sequence length, and conservation was given in Additional file 2_sheet 1 (human), 2 (mouse), and 3 (chicken). The distributions of AMFEs for the conserved and the non-conserved pre-miRNAs are shown in Figure 1. AMFEs in conserved and non-conserved pre-miRNA groups were from normal distribution expect mouse *S_c_*^1 ^group (Additional file 3), and for each species we statistically compared the mean AMFE and AMFE variances of *S_n _*with *S_c_*^1^, *S_c_*^2^, and *S_c_*^3 ^(Table 1). For pre-miRNAs from the human and chicken genomes, the mean AMFEs of the non-conserved and conserved pre-miRNAs were not statistically different. For pre-miRNAs from the mouse genome, although the mean AMFEs of *S_c_*^1 ^and *S_c_*^2 ^were significantly larger than that of *S_n _*at the 0.05 FDR level, the mean AMFE difference decreased and became non-significant for the *S_c_*^3 ^pre-miRNAs. The mean AMFE of *S_c_*^3 ^was only 2.8 kcal/mol higher than that of *S_n_*, while the mean AMFE of *S_c_*^1 ^was 4.1 kcal/mol higher. Therefore, although pre-miRNAs are significantly more stable than other non-coding RNAs [8], the conserved and non-conserved pre-miRNAs had relatively similar mean AMFEs.

**Table 1:** AMFE Comparison between non-conserved and conserved pre-miRNAs from human, mouse, and chicken genomes

Species	Conservation	AMFE (kcal/mol)	***p***_**1**_	***p***_**2**_
Human	*S_n _*(132)	44.1 ± 14.1		
	*S_c_*^1 ^(60)	45.2 ± 9.98	5.4×10^-1^	*3.7×10^-3^
	*S_c_*^2 ^(66)	44.6 ± 7.06	7.8×10^-1^	*8.6×10^-9^
	*S_c_*^3 ^(33)	44.7 ± 6.36	7.5×10^-1^	*2.3×10^-6^

Mouse	*S_n _*(108)	41.0 ± 9.79		
	*S_c_*^1 ^(63)	45.1 ± 7.83	*5.2×10^-3^	*4.5×10^-2^
	*S_c_*^2 ^(75)	45.0 ± 6.93	*2.4×10^-3^	*1.4×10^-4^
	*S_c_*^3 ^(39)	43.8 ± 5.89	5.6×10^-2^	*4.8×10^-4^

Chicken^a^	*S_n _*(21)	42.2 ± 14.7		
	*S_c_*^2 ^(40)	41.6 ± 5.59	8.7×10^-1^	*3.3×10^-7^
	*S_c_*^3 ^(19)	42.3 ± 5.05	9.8×10^-1^	*3.1×10^-5^

The number of pre-miRNAs within each set is indicated in brackets behind set name. ^a ^*S_c_*^1 ^for chicken was excluded as it contained fewer than five pre-miRNAs. AMFE, the AMFE mean values (± SD). Pair-wise comparisons were performed between non-conserved (*S_n_*) and conserved pre-miRNAs (*S_c_*^1^, *S_c_*^2^, and *S_c_*^3^) from human, mouse, and chicken genomes. *p*_1_, *P*-value of two-sample two-sided *t*-test (without assuming equal variance) for AMFE means. *p*_2_, *P*-value of two-sample two-sided *F*-test for AMFE variances. * the *P*-value is significant at the 0.05 FDR level.

Unlike the mean AMFE values, the AMFE variances of the conserved and non-conserved pre-miRNAs were significantly different in all three species at the FDR 0.05 level (Table 1). Moreover, the AMFE variance consistently decreased from *S_c_*^1 ^to *S*_c_^3 ^in all three species (in chicken *S_c_*^1 ^was excluded). The AMFE standard deviations of *S_n _*were 2.21-fold, 1.66-fold, and 2.91-fold larger than *S_c_*^3 ^for pre-miRNAs from the human, mouse, and chicken genomes, respectively.

Conserved and non-conserved pre-miRNAs were classed as relatively ultra stable or less stable to further investigate the stability distribution of pre-miRNAs. In each species, pre-miRNAs with AMFEs in the top 10 percent were classed as ultra stable, and in the bottom 10 percent were classed as unstable. In the *S_c_*^3 ^group, 3.0% (human), 2.6% (mouse), and 0.0% (chicken) of pre-miRNAs were ultra stable. In comparison, in the *S_n _*group, 16.7% (human), 9.3% (mouse), and 23.8% (chicken) were ultra stable. Similar results were obtained for unstable pre-miRNAs. In the *S_c_*^3 ^group, 3.0% (human), 2.6% (mouse), and 5.2% (chicken) of pre-miRNAs were unstable, while in the *S_n _*group, 18.2% (human), 19.4% (mouse), and 23.8% (chicken) of pre-miRNA were unstable. In summary, the mean AMFEs of conserved and non-conserved pre-miRNA AMFEs were similar, but the distribution of AMFEs for conserved pre-miRNAs was significantly smaller than that of non-conserved pre-miRNAs.

### Sequence comparison between conserved and non-conserved pre-miRNAs

We next investigated how the pre-miRNA nucleotide compositions were correlated with sequence conservation. Firstly, for all pre-miRNAs, we calculated the proportion of nucleotides forming base pairs (bp %) and the proportion of these base pairs that were A-U base pairs ((A-U) %) (Table 2, Additional file 4, see also Additional file 3 for normality test results). On average, the conserved pre-miRNAs contained significantly more base pairs (72.4%) than the non-conserved pre-miRNAs (68.3%, *p *= 8.7 × 10^-14^). However, the mean values of (A-U) % for the conserved pre-miRNAs were also significantly larger than those of the non-conserved pre-miRNAs (at 0.01 FDR level, in human and chicken; at 0.05 FDR level, mouse). On average, 49.2% (human), 48.6% (mouse), and 51.8% (chicken) base pairs of the conserved pre-miRNAs were A-U, compared with 42.7% (human), 44.4% (mouse), and 37.1% (chicken) of the non-conserved pre-miRNAs. In this study, conserved and non-conserved pre-miRNAs had similar mean AMFEs. This is consistent with the base pairing results as A-U base pairs are less stable than G-C base pairs. So, although the conserved pre-miRNAs have a higher bp % than the non-conserved pre-miRNAs, this is offset by a larger proportion of A-U base pairs. On the other hand, the distribution variances of both bp % and (A-U) % could explain the observed stability variances. Conserved pre-miRNAs had consistently smaller bp % and (A-U) % variances than non-conserved pre-miRNAs.

**Table 2:** Summary and comparison of pre-miRNA base pairing and nucleotide composition

Species	Conservation	bp %	(A-U) %	(A+U) %	A %	U %	G %	C %
Human	*S_n _*(132)	68.66 ± 7.83	42.73 ± 16.12	47.11 ± 13.18	22.10 ± 7.89	25.01 ± 6.96	27.49 ± 7.90	25.40 ± 7.24
	*S_c_*^1 ^(60)	71.88 ± 6.20^A^	49.34 ± 11.81^Ab^	53.87 ± 10.66^A^	23.78 ± 6.36	30.09 ± 6.42^A^	24.45 ± 5.53^AB^	21.68 ± 5.84^A^
	*S_c_*^2 ^(66)	72.94 ± 5.64^Ab^	48.67 ± 8.17^AB^	51.62 ± 7.15^AB^	23.41 ± 4.74^B^	28.21 ± 4.42^AB^	25.71 ± 3.49^aB^	22.67 ± 5.11^AB^
	*S_c_*^3 ^(33)	73.48 ± 4.67^AB^	50.14 ± 11.02^Ab^	52.94 ± 9.67^a^	22.96 ± 6.05	29.98 ± 6.16^A^	26.06 ± 5.15^B^	20.99 ± 5.92^A^

Mouse	*S_n _*(108)	67.87 ± 6.51	44.35 ± 11.20	49.30 ± 9.65	22.40 ± 6.16	26.90 ± 5.97	27.28 ± 5.57	23.42 ± 5.74
	*S_c_*^1 ^(63)	72.53 ± 5.85^A^	48.20 ± 11.47^a^	53.07 ± 9.42a	23.29 ± 5.42	29.78 ± 6.11^A^	25.30 ± 5.28^a^	21.62 ± 5.16^a^
	*S_c_*^2 ^(75)	72.10 ± 5.87^A^	47.70 ± 8.14^aB^	50.57 ± 6.77^B^	22.90 ± 4.57^b^	27.67 ± 4.40^b^	26.28 ± 3.72^B^	23.15 ± 5.02
	*S_c_*^3 ^(39)	73.37 ± 5.13^A^	50.85 ± 10.93^A^	53.05 ± 9.15a	22.89 ± 5.90	30.16 ± 5.51^A^	26.06 ± 5.06	20.90 ± 5.53^a^

Chicken*	*S_n _*(21)	68.24 ± 7.37	37.13 ± 17.43	44.17 ± 14.75	19.47 ± 8.79	24.69 ± 8.01	29.35 ± 6.00	26.48 ± 9.74
	*S_c_*^2 ^(40)	70.89 ± 7.06	51.22 ± 7.19^AB^	55.11 ± 4.99^AB^	24.60 ± 4.14^B^	30.52 ± 3.97^AB^	24.80 ± 3.53^AB^	20.08 ± 3.24^aB^
	*S_c_*^3 ^(19)	73.08 ± 5.14^a^	53.80 ± 7.56^AB^	57.18 ± 5.72^AB^	24.45 ± 5.26	32.74 ± 3.26^AB^	23.94 ± 4.49^A^	18.87 ± 3.33^AB^

Results represent the mean (± SD) of detailed information of base pairing and nucleotide composition. The number of pre-miRNAs within each set is indicated in brackets behind set name. * *S_c_*^1 ^for chicken was excluded as it contained fewer than five pre-miRNAs. Pair-wise comparisons of means (two-sided *t*-test) and variances (*F*-test) were performed between non-conserved (*S_n_*) and conserved pre-miRNAs (*S_c_*^1^, *S_c_*^2^, and S*_c_*^3^) from human, mouse, and chicken genomes. ^a ^mean value significantly different from that of *S_n _*at the 0.05 FDR level. ^A ^mean value is significantly different from that of *S_n _*at the 0.01 FDR level. ^b ^variance is significantly different from that of *S_n _*at the 0.05 FDR level. ^B ^variance significantly different from that of *S_n _*at the 0.01 FDR level.

**Figure 2:**
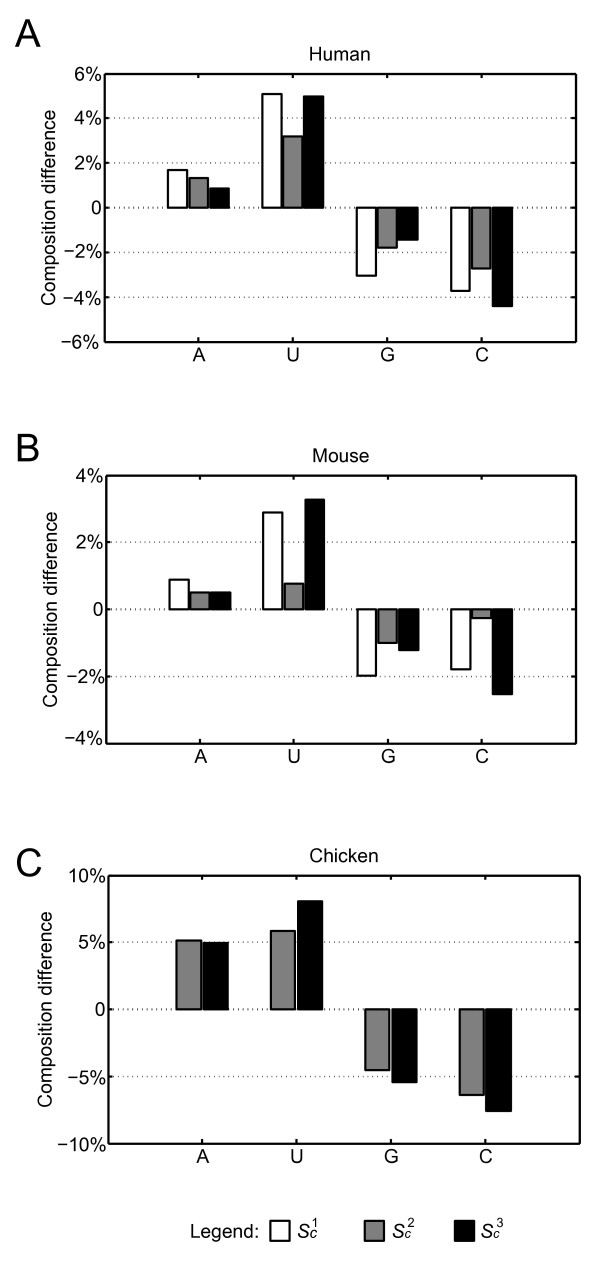
**Nucleotide composition difference between conserved and non-conserved pre-miRNAs**. The A, U, G, and C composition difference between conserved (*S_c_*^1^, *S_c_*^2^, and *S_c_*^3^) and non-conserved pre-miRNAs (*S_n_*) from human (A), mouse (B), and chicken (C) genomes. The white, gray, and black bars respectively denote the values that *S_c_*^1^, *S_c_*^2^, and *S_c_*^3 ^mean nucleotide compositions minus that of S*_n_. S_c_*^1 ^for chicken was excluded as it contained fewer than five pre-miRNAs.

The nucleotide composition of the conserved and non-conserved pre-miRNAs was also examined, as shown in Table 2 (see also Additional file 3 for normality test results). All conserved pre-miRNA sets had higher average A+U contents than G+C contents, while the A+U contents of the non-conserved pre-miRNA sets were all below 50% and significantly smaller than the conserved pre-miRNA sets, expect for the mouse *S_c_*^2 ^set. Compared with non-conserved pre-miRNAs, conserved pre-miRNAs had an increase of A and U contents but a decrease of G and C contents (Figure 2). Notably, the U content of conserved pre-miRNA was higher than A, G, and C. The average U content in overall conserved pre-miRNAs was 29.4% while the average A content was 23.4%. Furthermore, conserved pre-miRNAs had 3.4% higher U contents than non-conserved pre-miRNAs while the A content was only 1.4% higher in conserved pre-miRNAs than in non-conserved pre-miRNAs. In contrast, the G and C contents were 3.0% and 2.1% lower in conserved pre-miRNAs than in non-conserved pre-miRNAs respectively. We also observed that the nucleotide content of conserved pre-miRNAs has a narrower distribution than non-conserved pre-miRNAs.

### Structural element comparison between conserved and non-conserved pre-miRNAs

**Figure 3:**
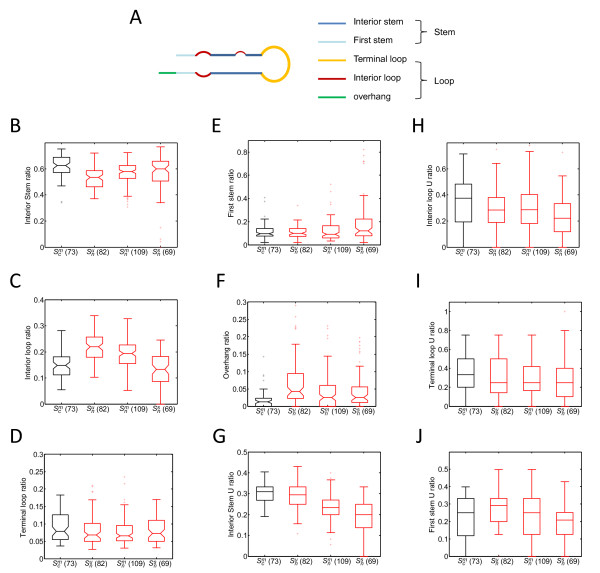
**Structural element features of conserved and non-conserved pre-miRNAs with different level of stability**. **(A) **diagram of structural elements of a hairpin secondary structure. **(B-F) **Boxplot (box with notch) of length ratios of interior stem (B), interior loop (C), terminal loop (D), first stem (E), and overhang (F) with respect to pre-miRNAs within *S_c_^m ^*(black), *S_n_^u ^*(red), *S_n_^m ^*(red), and *S_n_^s ^*(red). **(F-J) **Boxplot (box without notch) of region U ratios of interior stem (G), interior loop (H), terminal loop (I), and first stem (J) of pre-miRNAs within *S_c_^m^*, *S_n_^u^*, *S_n_^m^*, and *S_n_^s^*.

Hairpin secondary structures can be divided into five basic structural elements [24], including two kinds of stems (interior and first stem) and three kinds of loops (terminal, interior and overhang loops) (Figure 3A). We examined whether conserved and non-conserved pre-miRNAs differed with respect to structural elements. We compared non-conserved pre-miRNAs (*n *= 260, *S_n_*) with the most conserved pre-miRNAs (*n *= 91, *S_c_*^3^) from the three genomes, focusing on two features: (1) the ratio of the structural element length to the complete sequence length and (2) the ratio of U nucleotides to the total number of nucleotides within the structural element. Two AMFE thresholds were selected (*T*_1 _= 38.6 kcal/mol and *T*_2 _= 48.6 kcal/mol) corresponding to the 10^th ^quantile and 90^th ^AMFE quantile of the *S_c_*^3 ^pre-miRNAs respectively. Non-conserved pre-miRNAs were denoted as *S_n_^u^*, *S_n_^m^*, and *S_n_^s ^*with AMFEs <*T*_1_, *T*_1 _≤ AMFEs ≤ *T*_2_, and AMFEs >*T*_2_, respectively. *S_c_^m ^*denotes the conserved pre-miRNAs with AMFEs between *T*_1 _and *T*_2 _(80% of the total conserved pre-miRNAs).

Although AMFEs for pre-miRNAs in the *S_c_^m ^*and *S_n_^m ^*groups were comparable (Additional file 5), there was considerable variation in structural elements (Figure 3B-F). For the non-conserved pre-miRNAs, interior stem and first stem length ratios increased with increasing sequence stability. Surprisingly, the *S_c_^m ^*group exhibited significantly larger interior stem ratios on average than the *S_n_^s ^*group (*p *= 2.5 × 10^-5^). The *S_n_^m ^*group had smaller overhangs than both the *S_n_^u ^*and *S_n_^s ^*groups, but the overhangs of the *S_c_^m ^*group were even significantly smaller than those of the *S_n_^m ^*group (*p *= 3.2 × 10^-4^). The *S_c_^m ^*group also had significantly lower interior loop ratios (*p *= 3.2 × 10^-7^) and larger terminal loop ratios than the *S_n_^m ^*group (*p *= 4.4 × 10^-2^). Only first stem ratios of the *S_c_^m ^*and *S_n_^m ^*groups were comparable. On the other hand, the structural element U contents of conserved and non-conserved pre-miRNAs are shown in Figure 3G-J. The *S_c_^m ^*group had significantly higher U ratios at interior stem region (*p *= 3.1 × 10^-3^) than the *S_n_^m ^*group. The *S_c_^m ^*group also had higher U content on average than the *S_n_^u ^*group at terminal and interior loops, although the differences were not significant. No apparent increase of U content in the *S_c_^m ^*group first stem regions was observed. We did not compare U contents of overhangs due to their usually short element lengths.

## Discussion

Here we present a systematic comparison of structural stability for non-conserved and conserved pre-miRNAs from human, mouse, and chicken genomes. Previous studies have compared comparisons between other kinds of RNAs and native pre-miRNAs, and have proposed that pre-miRNAs are more stable [6,8]. Our results from comparisons within the pre-miRNA population provide novel insights into pre-miRNA thermodynamic stability and possible links with the pre-miRNA evolutionary process in animal species. The results presented here indicated both an upper and lower limit for pre-miRNA thermodynamic stability, implying a natural selection pressure against both ultra stable and unstable pre-miRNAs.

Moderately stable pre-miRNAs in animal could result from a trade-off between structural rigidity and flexibility. It is known that secondary structures of pre-miRNAs are needed for a correct recognition of specific enzymes in the miRNA biogenesis [2-5]. Thus maintaining a stable secondary structure could be necessary for pre-miRNA functioning, which could explain the result that unstable pre-miRNAs were less favoured by natural selection. However, the process of miRNA maturing also involves cleavage and duplex unwinding of pre-miRNAs [1]. Human Drosha has been reported to selectively cleave pre-miRNA with large terminal loop [18], which was consistent with our observation that conserved pre-miRNAs had on average larger terminal loops than non-conserved pre-miRNAs. It is also known that duplex unwinding is critical for the processing of pre-miRNAs to generate mature miRNAs [13,14,16]. As A-U base pairs were less stable than G-C base pairs, larger (A-U) % of conserved pre-miRNA could increase structural flexibility that facilitate the unwinding process. Conserved pre-miRNAs also had larger bp % than non-conserved pre-miRNAs, which could be possibly ascribed to (1) the influence of bp % on duplex unwinding was minor and/or (2) a trade-off between structure rigidity and flexibility.

However, the natural selection pressure for pre-miRNA stability could also involve selection for pre-miRNA characteristics other than thermodynamic stability, but that affect pre-miRNA stability as a side effect. We have shown that conserved and non-conserved pre-miRNAs with comparable AMFEs exhibited significant differences in structural elements. These differences might be due to a trade-off between pre-miRNA structural rigidity and flexibility, but the possibility of selection for factors other than thermodynamics could not be ignored.

In this study, the enrichment of U nucleotides in conserved pre-miRNAs was particularly noteworthy. High pre-miRNA U nucleotide content might both contribute to maintaining moderate stability and serve as a signal for miRNA biogenesis [8]. These results provide topics for future experimental and theoretical investigations, and raise an interesting theoretical question about the evolutionary dynamics underlying pre-miRNA structure and stability. Is the enrichment of U nucleotides in pre-miRNA the result of step-wise mutation accumulations or filtering from non-conserved pre-miRNAs? Exploration of this question could provide a deeper understanding of the miRNA evolutionary process and underlying mechanism.

As determining the conservation level of pre-miRNA sequences was critical for our analyses, we chose three genomes with abundant pre-miRNAs and used dual conservation constraints to select pre-miRNAs in this study. Although this method convincingly determined pre-miRNA conservation, it also reduced the size of the pre-miRNA population used for our investigation. 1,779 sequences of pre-miRNAs were obtained from human, mouse, and chicken genomes, from which 658 were selected for further analyses. As more pre-miRNAs are identified and their sequence conservation determined, the size of the pre-miRNA population available for study will increase, allowing for the identification of stronger general trends in the future. For instance, investigation of exhaustive miRNA families would allow us to derive the pre-miRNA evolutionary trajectory by comparing their thermodynamic stability, nucleotide compositions, structural features, and mutations from consensus ancestor sequences.

The results presented here might also be used in the future to predict or verify pre-miRNA candidates. The correlation between pre-miRNA thermodynamic stability and sequence conservation could be helpful for establishing more comprehensive pre-miRNA filtering criteria in practical applications. For instance, a loose candidate sequence filtering constraint could be applied to identify novel non-conserved pre-miRNAs for a given genome. On the contrary, a strict constraint for both unstable and ultra stable secondary structures would reduce the false positive rate for identifying novel conserved pre-miRNAs.

## Conclusions

In summary, our findings further the understanding of pre-miRNA thermodynamics, and might facilitate the investigation on miRNA evolution process. A correlation was identified between sequence conservation and thermodynamic stability for pre-miRNAs from human, mouse, and chicken genomes. The distribution of AMFEs for non-conserved pre-miRNAs was significantly larger than for conserved pre-miRNAs but the overall mean AMFEs of the two groups were similar. Investigation of pre-miRNA sequence features was used to explain their stability distribution. Compared with non-conserved pre-miRNAs, conserved pre-miRNAs form more base pairs on average but have a greater proportion of A-U bonds. Furthermore, the variances of sequence features of conserved pre-miRNAs, such as bp % and nucleotide composition, were consistently narrower than those for non-conserved pre-miRNAs. Notably, the U content of conserved pre-miRNAs was higher than the A, G, or C content, while the non-conserved pre-miRNAs had more G and C nucleotides, implying an importance of U nucleotide in pre-miRNA evolutionary history.

In addition to thermodynamic stability, we identified characteristic structural element features of conserved pre-miRNAs by comparing conserved and non-conserved pre-miRNAs with comparable stabilities. The results of this comparison indicated that the natural selection of pre-miRNA structure and sequence involved more than thermodynamic stability, indicating that pre-miRNAs evolutionary is a complex process.

## Methods

### Data

721, 579, and 479 pre-miRNA sequences of human (*Homo sapiens*), mouse (*Mus musculus*), and chicken (*Gallus gallus*) genomes respectively were downloaded from miRBase (Release 14) [25]. The genome assemblies for the pre-miRNA coordinates from human, mouse, and chicken genomes are GRCh37 (Feb 2009, hg19), NCBIM37 (July 2007, mm9), and WASHUC2 (May 2006, galGal3), respectively.

### Pre-miRNA structural stability

The program RNAfold (Vienna RNA package, version 1.7) was utilized with default parameter values to obtain estimated folding MFEs of pre-miRNAs [26,27]. To compare the stability of pre-miRNAs with different nucleotide sequence lengths, we used adjusted MFE (AMFE) [8]. AMFE is defined as AMFE = -MFE/(sequence length) × 100. Thus, pre-miRNAs with larger AMFE values were considered more stable.

### Pre-miRNA sequence conservation

The conservation level of pre-miRNA sequences was determined using two constraints. The first constraint used was University of California Santa Cruz PhastCons scores, which measure conservation for each nucleotide in a specific genome based on a phylogenetic hidden Markov model in a multiple alignment, for a given a phylogenetic tree [28,29]. Genomes of human (GRCh37), mouse (NCBIM37), and chicken (WASHUC2) were aligned against 44, 28, and 5 vertebrate genomes respectively to generate PhastCons scores. A pre-miRNA sequence was considered conserved if the average PhastCons scores in any 15-nucleotide sequence in the hairpin stem region were no smaller than 0.9 as described by Bentwich et al [19]. A few pre-miRNAs with non-hairpin structure were disregarded.

To reduce the false positive rate, the conservation of pre-miRNAs was checked using miRNA family classification in miRBase. The miRNA family classifications were produced by a BLAST-based clustering of all pre-miRNAs in the database followed by manual curation. The miRNA family classification provides information about pre-miRNA homologs. In each species, we filtered conserved pre-miRNAs, as defined by PhastCons scores, with few homologs and non-conserved pre-miRNAs with homologs in unrelated species. We also grouped the conserved pre-miRNAs into different sets according to the width of their homolog distribution in the phylogenetic tree. *M *was used to denote the number of taxonomic families where a given miRNA family was distributed. Conserved pre-miRNAs from miRNA families with an *M *< 5 and non-conserved pre-miRNA from families with an *M *> 1 were excluded from the study population. *S_n _*was used to denote the non-conserved pre-miRNA set containing non-conserved pre-miRNAs from families with *M *= 1. As the *M *values of miRNA families varied largely, we grouped conserved pre-miRNAs into three sets, *S_c_^i^*, *i *= 1, 2, and 3, that contain conserved pre-miRNAs from miRNA families with *M *values of 5 - 9, 10 - 19, and ≥ 20, respectively.

### Statistical tests

Pair-wise comparisons were performed for conserved and non-conserved pre-miRNAs. A two-sample, two-sided *t*-test without assumption of equal variance was used to compare the mean AMFE, nucleotide composition, and other characteristics. A two-sample two-sided *F*-test was applied to compare the distribution variances. A Lilliefors test [30] was used for testing normality. For the *P*-values produced by pair-wise comparison of a given characteristics between non-conserved and conserved pre-miRNAs, False Discovery Rate (FDR) controlling with Benjamini Hochberg procedure [31] was used for the multiple-testing correction.

## Authors' contributions

MN is the major researcher and prepared the manuscript. WS and XB were involved in data analyses and helped to revise the manuscript. SW and SL participated in discussion and guided the project. All authors read and approved the final manuscript.

## Supplementary Material

Additional file 1 **Figure S1**. Correlation between pre-miRNA sequence length and MFE values (A) as well as AMFE values (B). Dashed line is the linear regression of MFEs with sequence lengths. Click here for file

Additional file 2 **List of pre-miRNA name, length, AMFEs, and conservation level**. This file lists names, lengths, AMFEs, and conservation level of the pre-miRNAs we studied from human, mouse, and chicken genomes. The conservation level of 0, 1, 2, or 3 represents the pre-miRNAs belonging to group *S_n_*, *S_c_*^1^, *S_c_*^2^, and S*_c_*^3^, respectively. Click here for file

Additional file 3 **Table S1**. *P*-values of Lilliefors test for normality [30]. For non-conserved (*S_n_*) and conserved pre-miRNAs (*S_c_*^1^, *S_c_*^2^, and S*_c_*^3^) from human, mouse, and chicken genomes, Lilliefors test was used to test normality of AMFE, bp %, (A-U) %, (A+U) %, A%, G%, C%, and U%. The *P*-values of test for overall pre-miRNAs from each genome was also given. * not a normal distribution at the 0.05 level. ** *S_c_*^1 ^for chicken was excluded as it contained fewer than five pre-miRNAs. Click here for file

Additional file 4 **Figure S2**. Distribution of bp % and (A-U) % values of non-conserved (*S_n_*) and conserved pre-miRNAs (*S_c_*^1^, *S_c_*^2^, and *S_c_*^3^) from human, mouse, and chicken genomes. The number of pre-miRNAs within each set is indicated in brackets. *S_c_*^1 ^for chicken was excluded as it contained fewer than five pre-miRNAs. Click here for file

Additional file 5 **Figure S3**. Distributions of AMFEs of pre-miRNAs within *S_c_^m^*, *S_n_^u^*, *S_n_^m^*, and *S_n_^s^*. Click here for file
