# Supplementary figures and images for: Correlation between sequence conservation and structural thermodynamics of microRNA precursors from human, mouse, and chicken genomes
Source: BMC Evol Biol. 2010 Oct 27;10:329. doi: 10.1186/1471-2148-10-329 (PMC2984420; doi:10.1186/1471-2148-10-329)

**A**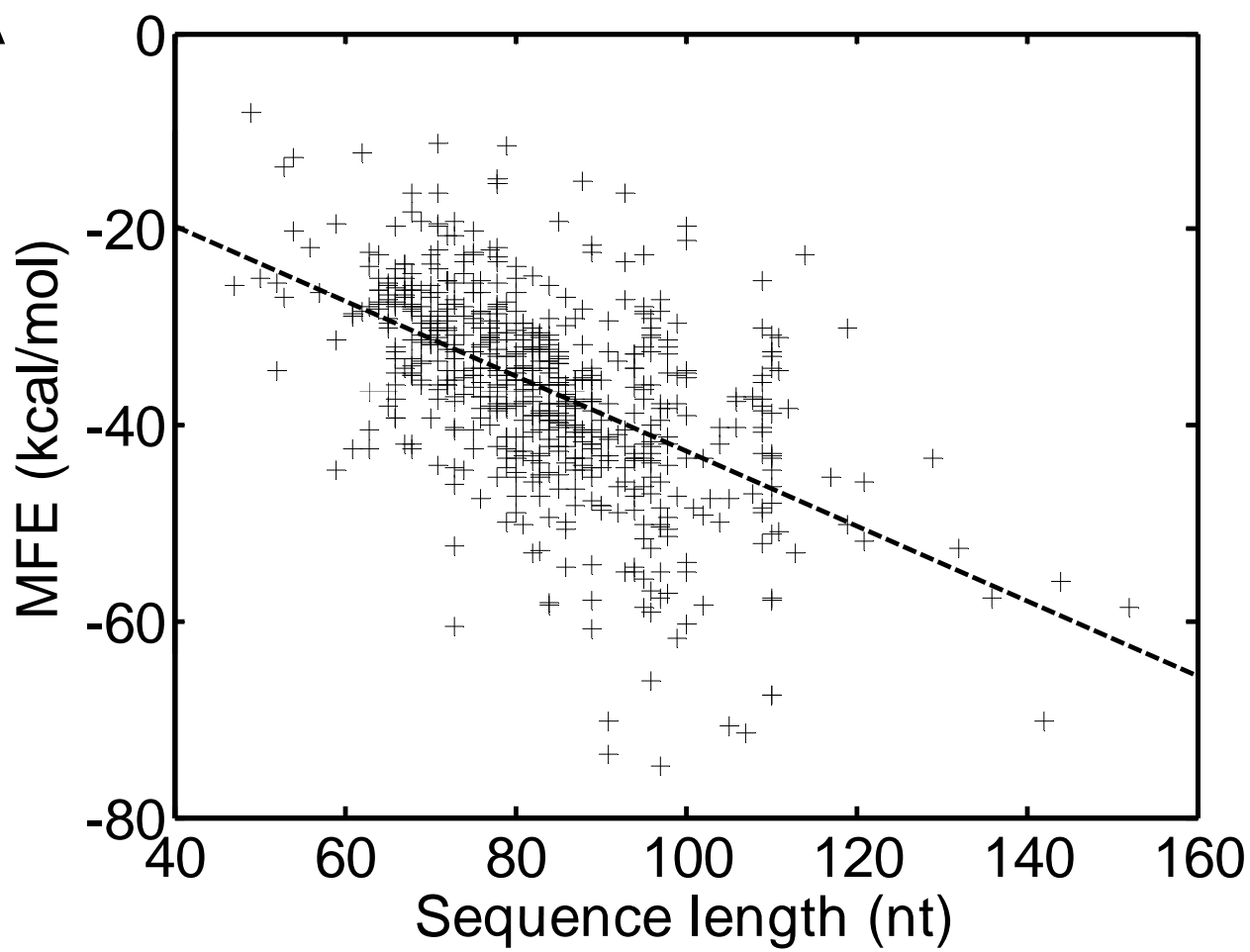**B**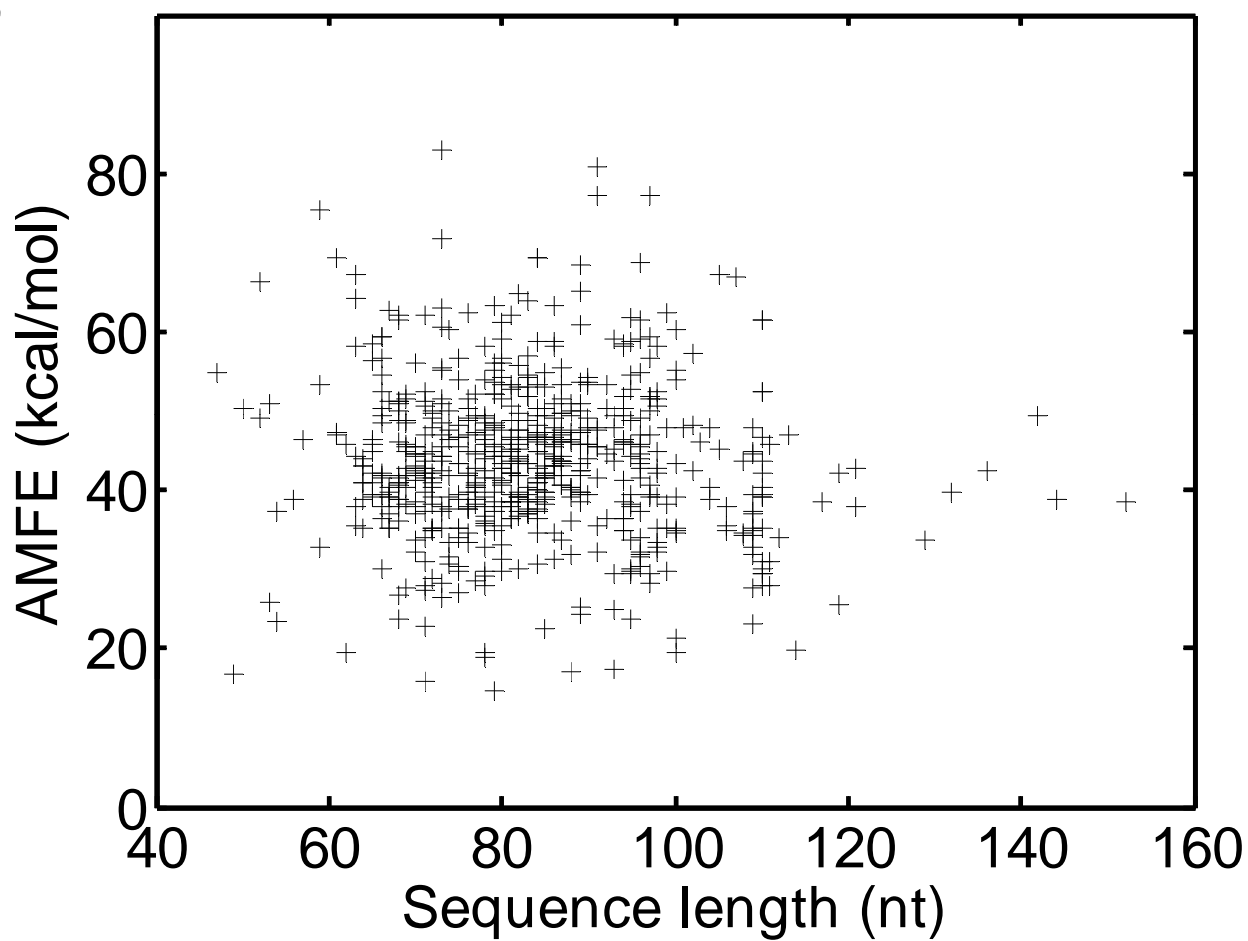

Supplement: Additional file 1 — Figure S1. Correlation between pre-miRNA sequence length and MFE values (A) as well as AMFE values (B). Dashed line is the linear regression of MFEs with sequence lengths. [file 1471-2148-10-329-S1.PDF]

Human

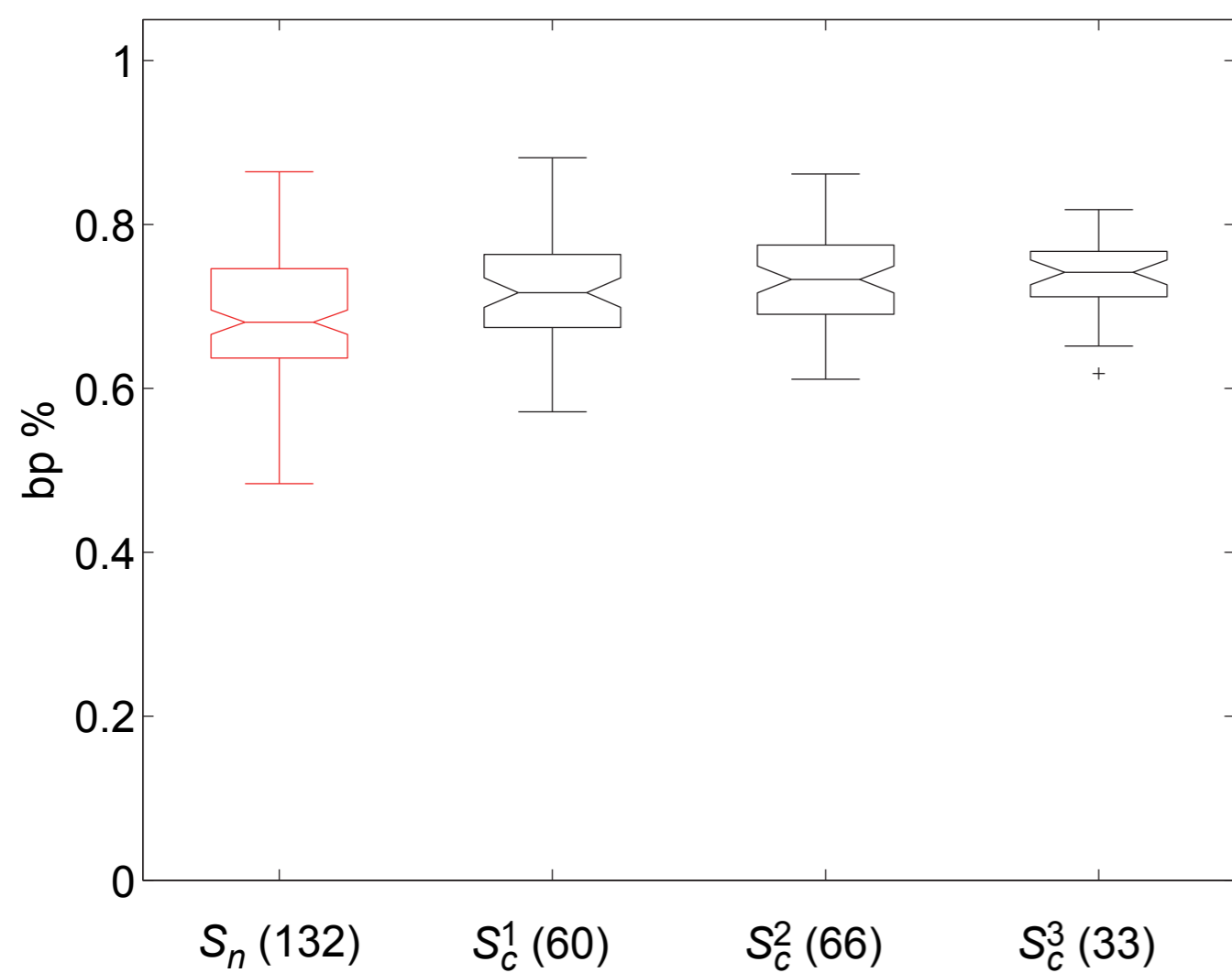

Human

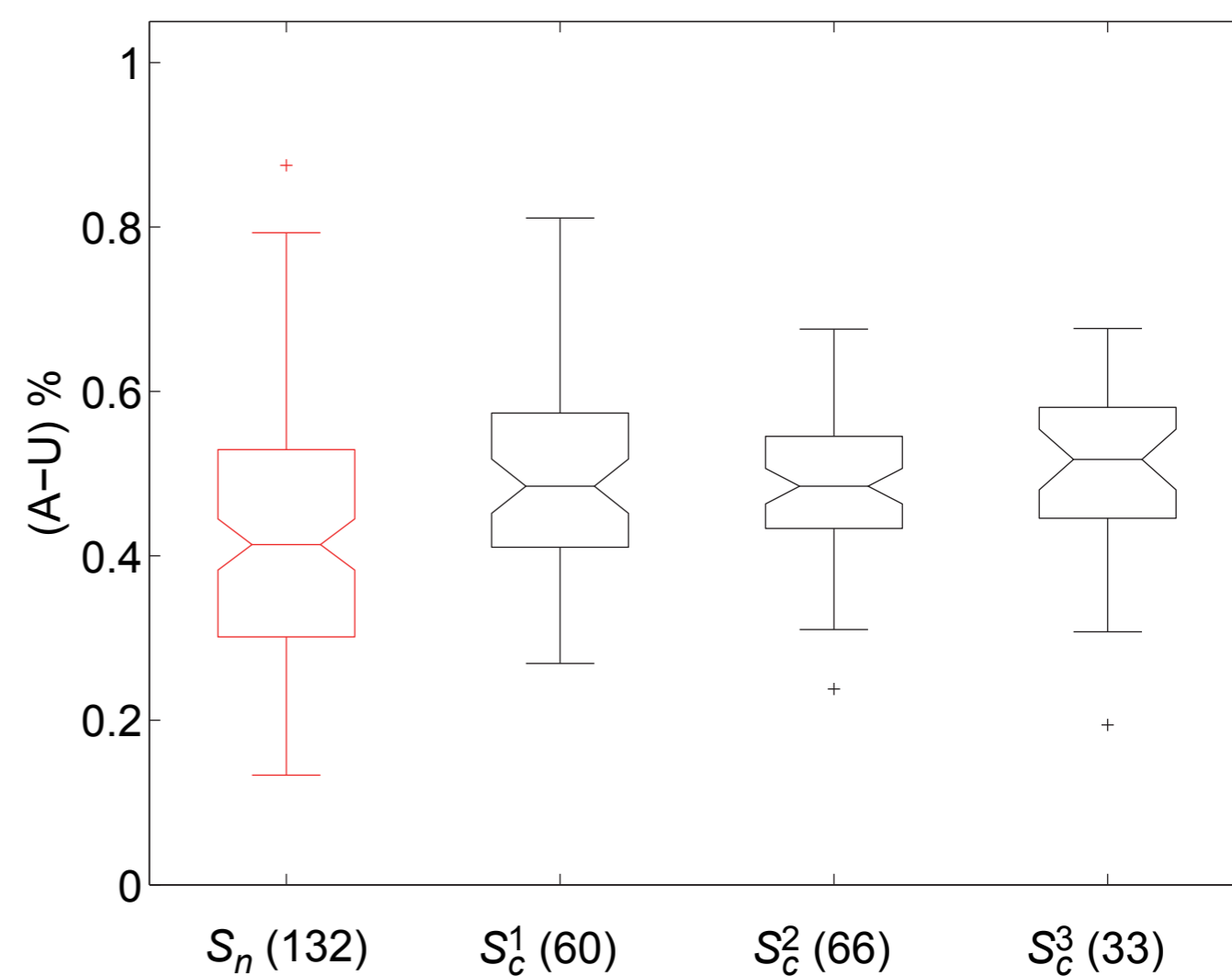

Mouse

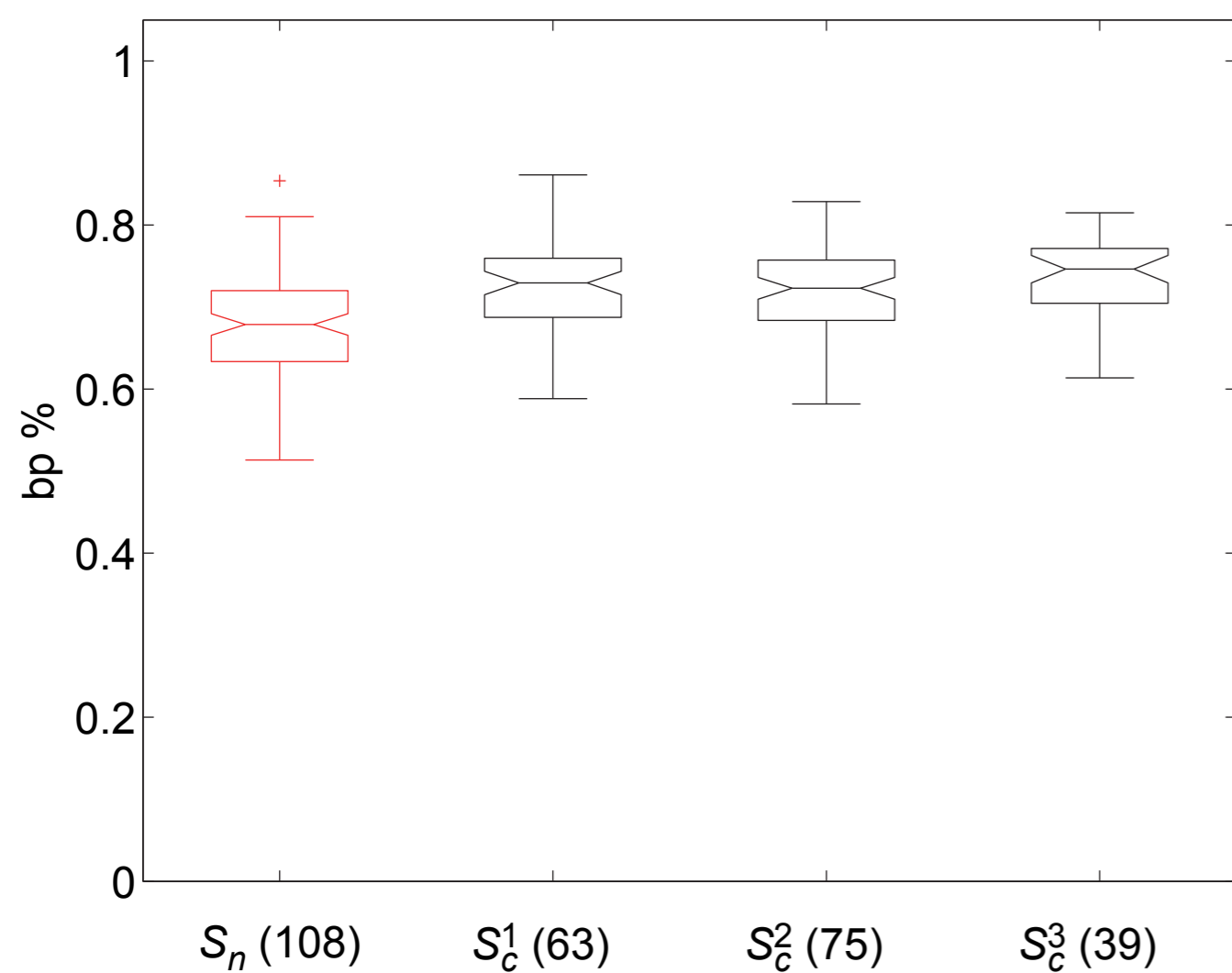

Mouse

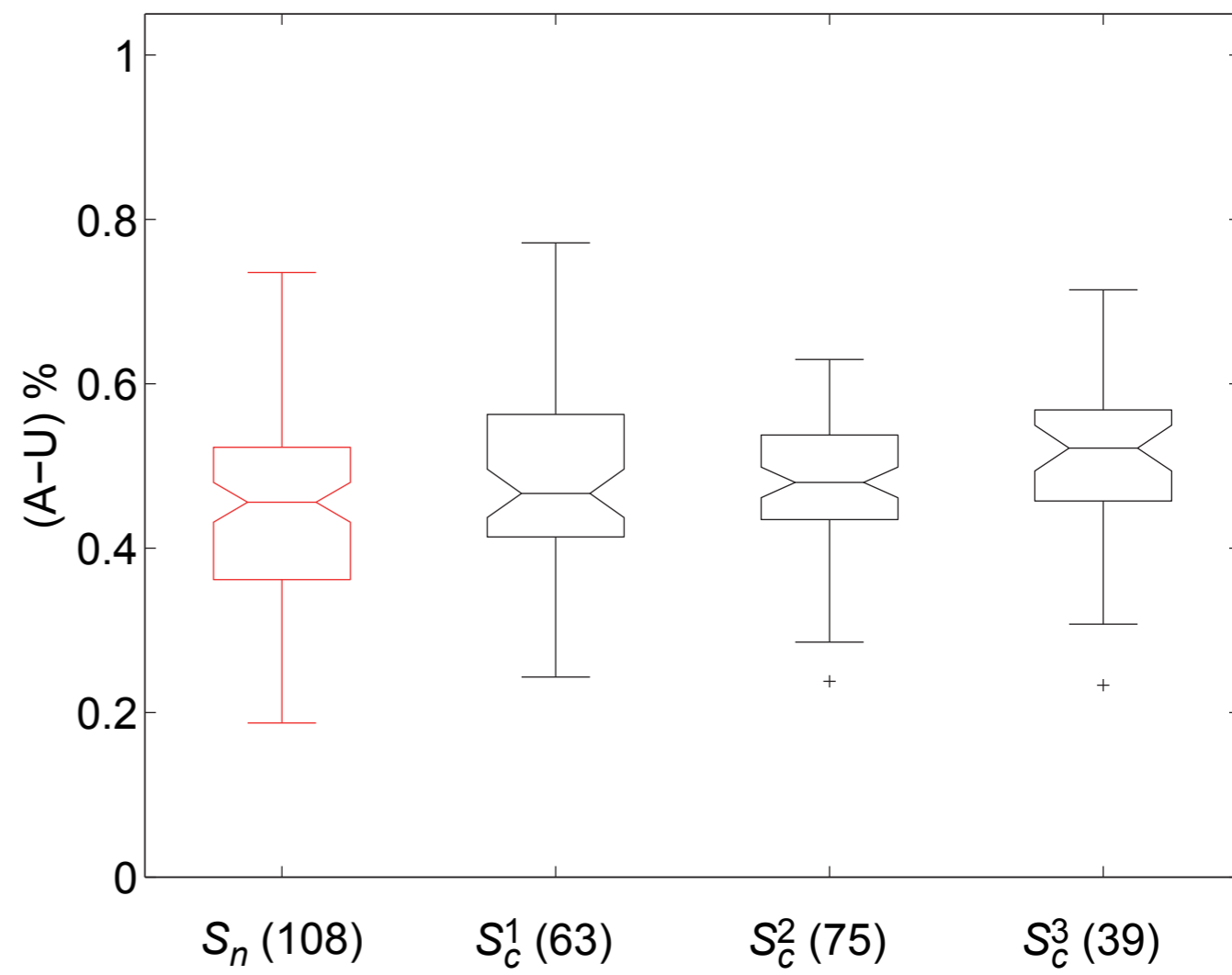

Chicken

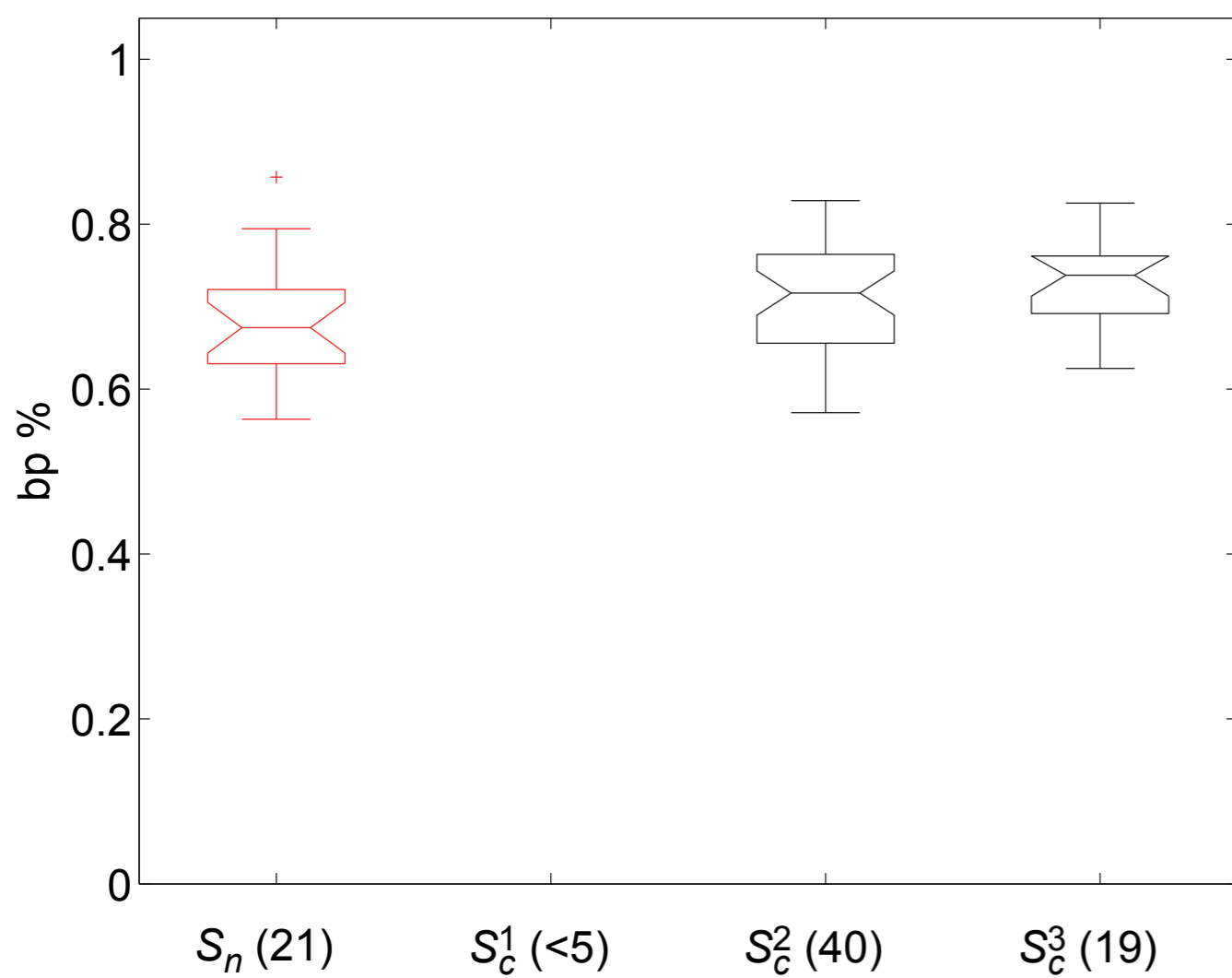

Chicken

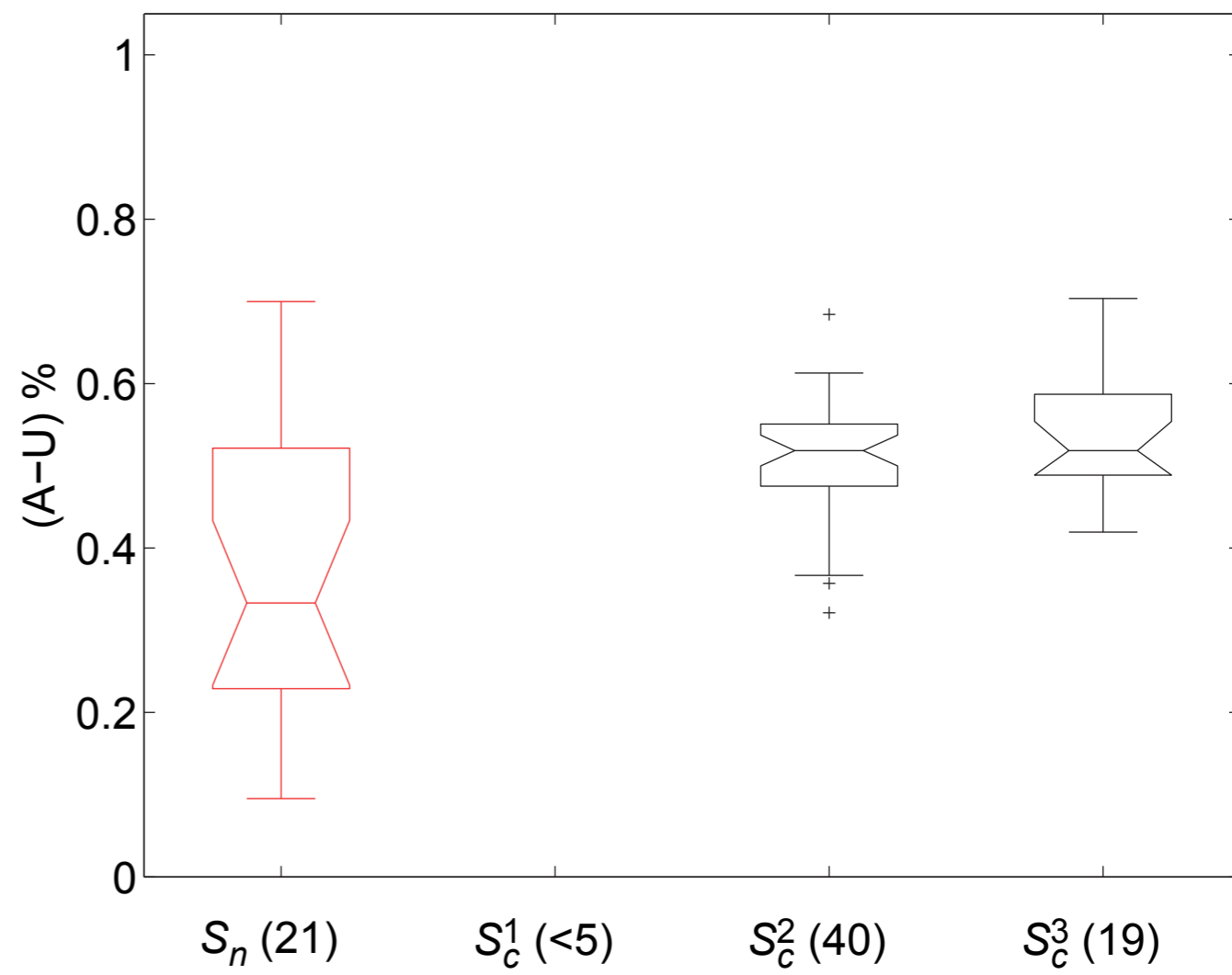

Supplement: Additional file 4 — Figure S2. Distribution of bp % and (A-U) % values of non-conserved (Sn) and conserved pre-miRNAs (Sc1, Sc2, and Sc3) from human, mouse, and chicken genomes. The number of pre-miRNAs within each set is indicated in brackets. Sc1 for chicken was excluded as it contained fewer than five pre-miRNAs. [file 1471-2148-10-329-S4.PDF]

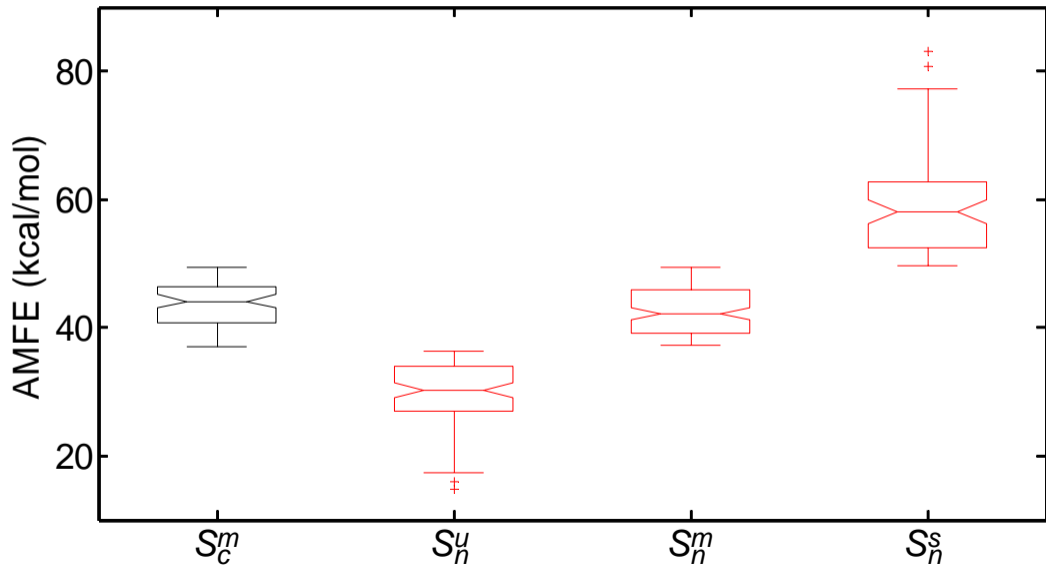

Supplement: Additional file 5 — Figure S3. Distributions of AMFEs of pre-miRNAs within Scm, Snu, Snm, and Sns. [file 1471-2148-10-329-S5.PDF]
