# Supplementary material for: Correlation between sequence conservation and structural thermodynamics of microRNA precursors from human, mouse, and chicken genomes
Source: BMC Evol Biol. 2010 Oct 27;10:329. doi: 10.1186/1471-2148-10-329 (PMC2984420; doi:10.1186/1471-2148-10-329)
Supplement: Additional file 3 — Table S1. P-values of Lilliefors test for normality [30]. For non-conserved (Sn) and conserved pre-miRNAs (Sc1, Sc2, and Sc3) from human, mouse, and chicken genomes, Lilliefors test was used to test normality of AMFE, bp %, (A-U) %, (A+U) %, A%, G%, C%, and U%. The P-values of test for overall pre-miRNAs from each genome was also given. * not a normal distribution at the 0.05 level. ** Sc1 for chicken was excluded as it contained fewer than five pre-miRNAs. [file 1471-2148-10-329-S3.PDF]

Table S1. *P*-values of Lilliefors test for normality

		All	S_n	S_c^{1**}	S_c^{2}	S_c^{3}
Human	AMFE	*0.046	> 0.5	> 0.5	0.46	0.054
	bp %	*0.002	> 0.5	0.054	0.29	0.053
	(A-U) %	0.064	> 0.5	> 0.5	> 0.5	> 0.26
	(A+U) %	*0.070	> 0.5	*0.021	0.11	0.28
	A %	> 0.5	0.32	0.27	0.14	0.17
	G %	0.015	> 0.5	> 0.5	> 0.5	> 0.5
	C %	*0.0013	> 0.5	> 0.5	0.11	0.069
	U %	0.32	> 0.5	0.48	0.41	> 0.5
Mouse	AMFE	*0.0028	*0.0073	0.34	0.10	> 0.5
	bp %	0.08	> 0.5	> 0.5	> 0.5	*0. 022
	(A-U) %	0.23	0.35	0.18	0.50	0.26
	(A+U) %	0.34	> 0.5	0.28	0.11	0.12
	A %	0.27	0.11	0.27	> 0.5	> 0.5
	G %	0.098	0.10	0.42	*0.0088	> 0.5
	C %	0.024	> 0.5	0.50	*0.037	0.20
	U %	> 0.5	0.44	0.39	> 0.5	> 0.5
Chicken	AMFE	*0.041	> 0.5	-	0.32	0.37
	bp %	0.20	> 0.5	-	0.36	> 0.5
	(A-U) %	*< 0.001	0.46	-	*0.0069	0.10
	(A+U) %	*< 0.001	> 0.5	-	0.30	> 0.5
	A %	0.21	> 0.5	-	> 0.5	> 0.5
	G %	0.081	0.23	-	0.47	> 0.5
	C %	*< 0.001	> 0.5	-	0.059	> 0.5
	U %	*0.0071	> 0.5	-	0.50	> 0.5

* not a normal distribution at the 0.05 level.

** S_c^{1} for chicken was excluded as it contained fewer than five pre-miRNAs.
